# House-level risk factors associated with the colonization of broiler flocks with *Campylobacter *spp. in Iceland, 2001 – 2004

**DOI:** 10.1186/1746-6148-3-30

**Published:** 2007-11-12

**Authors:** Michele T Guerin, Wayne Martin, Jarle Reiersen, Olaf Berke, Scott A McEwen, Jean-Robert Bisaillon, Ruff Lowman

**Affiliations:** 1 Department of Population Medicine, Ontario Veterinary College, University of Guelph, Guelph, Ontario, N1G 2W1, Canada; 2 Reykjagarður hf, Fosshals 1, 112 Reykjavík, Iceland; 3 Agricultural Agency of Iceland, Austurvegur 64, 800 Selfoss, Iceland; 4 Department of Biometry, Epidemiology and Information Processing, University of Veterinary Medicine Hannover, Bünteweg 2, D-30559 Hannover, Germany; 5 Canadian Food Inspection Agency, Ottawa, Ontario, K2H 8P9, Canada

## Abstract

**Background:**

The concurrent rise in consumption of fresh chicken meat and human campylobacteriosis in the late 1990's in Iceland led to a longitudinal study of the poultry industry to identify the means to decrease the frequency of broiler flock colonization with *Campylobacter*. Because horizontal transmission from the environment is thought to be the most likely source of *Campylobacter *to broilers, we aimed to identify broiler house characteristics and management practices associated with flock colonization. Between May 2001 and September 2004, pooled caecal samples were obtained from 1,425 flocks at slaughter and cultured for *Campylobacter*. Due to the strong seasonal variation in flock prevalence, analyses were restricted to a subset of 792 flocks raised during the four summer seasons. Logistic regression models with a farm random effect were used to analyse the association between flock *Campylobacter *status and house-level risk factors. A two-stage process was carried out. Variables were initially screened within major subsets: ventilation; roof and floor drainage; building quality, materials and repair; house structure; pest proofing; biosecurity; sanitation; and house size. Variables with p ≤ 0.15 were then offered to a comprehensive model. Multivariable analyses were used in both the screening stage (i.e. within each subset) and in the comprehensive model.

**Results:**

217 out of 792 flocks (27.4%) tested positive. Four significant risk factors were identified. *Campylobacter *colonization was predicted to increase when the flock was raised in a house with vertical (OR = 2.7), or vertical and horizontal (OR = 3.2) ventilation shafts, when the producer's boots were cleaned and disinfected prior to entering the broiler house (OR = 2.2), and when the house was cleaned with geothermal water (OR = 3.3).

**Conclusion:**

The increased risk associated with vertical ventilation shafts might be related to the height of the vents and the potential for vectors such as flies to gain access to the house, or, increased difficulty in accessing the vents for proper cleaning and disinfection. For newly constructed houses, horizontal ventilation systems could be considered. Boot dipping procedures should be examined on farms experiencing a high prevalence of *Campylobacter*. Although it remains unclear how geothermal water increases risk, further research is warranted to determine if it is a surrogate for environmental pressures or the microclimate of the farm and surrounding region.

## Background

*Campylobacter *spp. remain one of the most frequent bacterial causes of foodborne gastroenteritis world-wide [1]. Poultry, and specifically consumption of undercooked poultry and mishandling raw poultry, is an important source of *Campylobacter *to humans [2-7]. The prevalence of broiler flocks colonized with *Campylobacter *spp. varies, ranging from 5% of flocks to more than 90% [8]. Once a flock is exposed, the bacteria spread rapidly throughout the flock, and most of the birds become colonized and remain so until slaughter [9-14]. Due to the difficulties in eliminating contamination of carcasses in slaughter plants, the control of *Campylobacter *in broiler flocks and production of birds free from colonization at slaughter, is essential for preventing human cases [5,14-17].

Many researchers [11,12,14,15,18-28] have contested that the most likely source of *Campylobacter *to broiler flocks is the environment (i.e. horizontal transmission). Both *Campylobacter*-positive and -negative flocks can be present in different houses on the same farm during the same growing period [9,11,12], illustrating that it is possible to prevent *Campylobacter *from entering a broiler house. Thus, it might be hypothesized that certain characteristics of a broiler house, or management practices at the house-level, might influence the likelihood that a flock will be exposed to the bacteria. House-level factors associated with an increased risk of colonization include: concrete floors (compared to wood floors) [12]; feed dispenser in the anteroom (compared to in the chicken room) [12]; evidence of mice [12]; absence of, or ineffective, hygiene barrier or biosecurity measures [12,14,16,29-31]; roof fans (compared to side fans) [16,32]; a static ventilation system (versus a dynamic system) [33]; two or more persons taking care of the house [33]; non-cement floors [34]; and poor maintenance of house surroundings [34]. In Iceland, flock-level risk factors have been studied [32]; however, to date, the association between broiler flock *Campylobacter *status and the attributes and management practices of broiler houses in Iceland has not been studied.

The strong association between the increased incidence of human campylobacteriosis and increased consumption of fresh chicken meat in Iceland in the late 1990's prompted a longitudinal study of the poultry industry [35]. The ultimate goal of the full project was to identify the means to decrease the frequency of broiler flock colonization with *Campylobacter*, thereby reducing the burden of foodborne illness associated with poultry consumption. Our objective in this study was to identify house-level risk factors associated with the colonization of broiler flocks with *Campylobacter *spp. in Iceland.

## Results

### Descriptive summary

**Figure 1:**
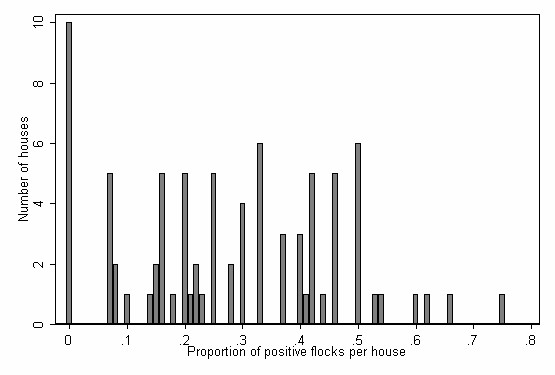
Distribution of the proportion of positive flocks among broiler houses in Iceland (n = 83 houses).

There were 792 flocks raised in the summer seasons, and of these, 217 (27.4%) tested positive for *Campylobacter*. The 792 flocks originated from 83 houses on 33 farms. The median number of flocks per house was 10 (mean 10, range 1 to 15). Ten houses did not have any positive flocks; the number of flocks raised in these houses ranged from 1 to 15, with the majority having ≥ 9 flocks per house. The distribution of the proportion of positive flocks per house is shown in Figure 1. Almost half of the house-level variables were consistent for all flocks raised in the same house during the study period. These included all of the drainage variables except for the floor drain method, heating of the broiler house floor, floor material, house water distribution system, sharing a common wall or entrance with another house, floor level, changing boots before entry to the house, use of geothermal water for cleaning, floor area, and cubic space. The median number of flocks per farm was 14 (mean 24, range 1 to 146). Three of 33 farms did not have any positive flocks; these were primarily the smaller farms that raised between 1 and 9 flocks.

Of the 217 positive flocks, 157 (72.4%) were slaughtered in 1 catch lot (for a total of 4 pooled samples per flock), 46 (21.2%) were slaughtered in 2 catch lots (for a total of 8 pooled samples per flock), and 14 (6.5%) were slaughtered in 3 or 4 catch lots (for a total of 12 to 16 pooled samples per flock). For flocks with more than one catch lot, the number of days between the first and last catch lot was relatively short (median 2, mean 2.5, range 1 to 59), and 82.2% of the flocks with multiple catch lots were positive in samples collected from the first catch lot. On the basis of catch lot sampling, out of 291 positive catch lots, 266 (91.4%) were positive in all 4 pooled samples, 2 (0.7%) were positive in 3 pooled samples, 6 (2.1%) were positive in 2 pooled samples, and 17 (5.8%) were positive in only 1 pooled sample.

### Statistical analysis

#### Random effects

The intra-class correlation coefficient (ICC) of a null model with only a house random effect was 0.10 (p < 0.001); the ICC of a null model with only a farm random effect was 0.21 (p < 0.001). When both random effects were included together in a null model, the variance of the farm random effect (0.6) was substantially higher than the variance of the house random effect (1.4 × 10^-15^). Based on these observations, a random effect at the farm level was included in all models to control for clustering.

### Subset analyses

Variable screening was carried out within subsets of potential risk factors; a separate multivariable analysis was used for each subset. In general, only one to three variables from each subset met the significance criterion of p ≤ 0.15 (Tables 1 and 2). The following predictors met this criterion and were made available to the comprehensive model: ventilation type; roof drain method; heating of broiler house floor; floor cracks fixed between flocks; ceiling; insect and rodent control; boots cleaned and disinfected before entry to the house; split room entry to the house; use of geothermal water for cleaning; and floor area. No significant interaction or quadratic terms were identified.

**Table 1:** House-level categorical variables available for analysis of *Campylobacter *colonization of broilers in Iceland

Variables within subsets	Description of variable or level	Number of negative flocks	Number of positive flocks
**Ventilation**			

Ventilation system – Regular cleaning^a ^(n = 699)	Yes (after each flock)	500	196
	Sometime (every other time on average)	2	1
Ventilation integrity (n = 758)	Closed (wild birds or their faeces are unable to enter the house)	354	142
	Open (wild birds or their faeces are able to enter the house)	198	64
Ventilation type^b ^(n = 758)	Both	153	80
	Horizontal	86	7
	Vertical	313	119

**Roof and floor drainage**			

Roof and floor drains merge^c ^(n = 758)	Roof drains are present & merge with floor drain	35	23
	Roof & floor drains do not merge or house does not have roof drains	517	183
Roof drain method^b ^(n = 758)	House does not have roof drains^d^	294	108
	Drains onto ground immediately outside the house^d^	129	45
	Drains into a ditch, trench, open area, field, or standing water near the house^d^	31	9
	Drains into water with a continuous flow	42	2
	Drains into a septic tank with overflow into an open trench	21	19
	Drains into a septic tank with overflow into an underground bed	35	23
Floor drains sealed (n = 758)	Yes	468	198
	No	84	8
Floor drain method (n = 758)	Drains onto ground immediately outside the house^d^	10	2
	Drains into a ditch, trench, open area, field, or standing water near the house^d^	28	12
	Drains into water with a continuous flow	117	45
	Drains into a septic tank with overflow into an open trench	108	61
	Drains into a septic tank with overflow into an underground bed	223	58
	Drains into sub-level of the house, then pumped out onto fields^d^	66	28

**Building quality, materials and repair**			

Heating of broiler house floor^b ^(n = 699)	Yes	172	106
	No	330	91
Quality of floor litter storage facility (n = 699)	Very good (above specification or needs)	307	114
	Good or average (meets specification or needs)^d^	187	76
	Inferior (clearly below specification or needs)^d^	8	7
Floor – material^a ^(n = 699)	Entirely concrete	496	194
	Includes wood elements	6	3
Floor – cracks fixed between flocks^b ^(n = 699)	Yes	231	100
	No	271	97
Wall material (within birds height) (n = 699)	Metal^d^	7	4
	Concrete^d^	420	160
	Wood	75	33
Ceiling^b ^(n = 699)	Metal	341	154
	Concrete	57	8
	Wood	104	35
House water distribution system (n = 699)	Nipples	378	150
	Other	124	47

**House structure**			

House shares a common wall with another house (n = 792)	Yes	268	94
	No	307	123
Floor level (n = 792)	Single-level house	546	199
	Multi-level house	29	18
House shares a common entrance with another house (n = 792)	Yes	179	47
	No	396	170

**Pest proofing**			

Insect and rodent control^b ^(n = 699)	Regular professional extermination	316	161
	Regular non-professional extermination	108	22
	No regular extermination	78	14
Construction pest proofing and sealing of house (n = 699)	Very good	386	163
	Average^d^	107	30
	Inferior^d^	9	4
Past evidence/observation of mice in house (n = 699)	Very rarely (< 2 observations per year)	487	193
	Sometime (1 to 3 observations per month)^d^	9	1
	Often (1 or more observations per week)^d^	6	3
Vegetation within 1 m of house (n = 699)	Yes	234	91
	No	268	106

**Biosecurity**			

Boots cleaned and disinfected before entry to house^b ^(n = 699)	Always	325	164
	Sometime (according to need)^d^	13	1
	Never^d^	164	32
Staff exclusively assigned to house (n = 699)	Yes	174	72
	No	328	125
Boots changed before entry to house^a ^(n = 699)	Yes	499	196
	No	3	1
Split room entry to house^b ^(n = 699)	Yes	284	145
	No	218	52

**Sanitation**			

Use of geothermal water for cleaning^b ^(n = 699)	Yes	336	175
	No	166	22
Use of chemical cleaner after clean-out and rinse^e ^(n = 699)	Yes	447	188
	No	55	9
Use of bacterial disinfectant and/or fumigation^e ^(n = 699)	Yes	463	193
	No	39	4

^a ^Variable excluded from the subset analysis due to the small number of observations (≤ 10) in one or more categories.

^b ^Variable retained after the subset analysis (p ≤ 0.15) for inclusion in the comprehensive model.

^c ^Variable excluded from the subset analysis due to high correlation (τ ≥ 0.8) with other variables (or categories of other variables) in the same subset.

^d ^Categories (denoted by ^d^) of a variable were combined for the subset analysis due to the small number of observations (≤ 15) in one or more categories, and/or to group biologically meaningful categories together.

^e ^Variable excluded from the subset analysis due to the occurrence of cells with very low frequencies of observations when the variables were in the model together.

**Table 2:** House-level continuous variables available for analysis of *Campylobacter *colonization of broilers in Iceland

Variables within subsets	Description	Mean	Median	Minimum	Maximum
**House size**					
Floor area^a ^(n = 792)	Floor area of broiler house (m^2^)	418	350	75	1089
Cubic space^b ^(n = 792)	Cubic area of broiler house (m^3^)	1028	890	187	2400

^a ^Variable retained after the subset analysis (p ≤ 0.15) for inclusion in the comprehensive model.

^b ^Variable excluded from the subset analysis due to high correlation (r ≥ 0.8) with another variable in the same subset.

### Comprehensive model

The final model contained four significant (p ≤ 0.05) predictors. The risk of *Campylobacter *colonization was predicted to increase when the flock was raised in a house with vertical (OR = 2.7), or vertical and horizontal (OR = 3.2) ventilation shafts, when the producer's boots were cleaned and disinfected prior to entering the broiler house (OR = 2.2), and when the house was cleaned with geothermal water (OR = 3.3) (Table 3). The distribution of these variables among broiler houses is shown in Table 4.

**Table 3:** Final logistic model^a ^for house-level factors associated with *Campylobacter *in broilers in Iceland (n = 675)

Variable	b	SE	p-value	95% CI (b)	OR	95% CI (OR)
Ventilation type						
Horizontal	Ref.	-	-	-	1.00	-
Both	1.15	0.44	0.008	0.30, 2.01	3.17	1.34, 7.47
Vertical	1.00	0.43	0.019	0.17, 1.84	2.73	1.18, 6.30
Boots cleaned and disinfected before entry to house	0.77	0.23	0.001	0.33, 1.21	2.16	1.39, 3.36
Use of geothermal water for cleaning house	1.18	0.25	< 0.001	0.68, 1.68	3.26	1.98, 5.35
Intercept	-3.41	0.45	< 0.001	-4.30, -2.52	-	-

^a^Log likelihood = -368.8, AIC = 749.7 Random farm effect: ρ = 2.5 × 10^-7^, Likelihood ratio test of ρ = 0: p = 1.000

**Table 4:** Distribution of selected variables among broiler houses in Iceland

Variable	Description of variable or level	Number of houses
Ventilation type (n = 75)	Both	25
	Horizontal	6
	Vertical	42
	Variable changed during the study period (from horizontal to vertical)	2
Boots cleaned and disinfected before entry to house (n = 80)	Always	54
	Sometime (according to need)	0
	Never	21
	Variable changed during the study period (from never to always)	2
	Variable changed during the study period (from sometime to never)	3
Use of geothermal water for cleaning (n = 80)	Yes	59
	No	21

### Model diagnostics

To assess the effect of performing model diagnostics on the final model without a random effect, we compared the parameter estimates of our final model to the same model without a random effect at the farm level, and found that the estimates remained stable. The ICC (ρ = 2.5 × 10^-7^, p = 1.000) of the final model was extremely small, indicating that there was very little clustering at the farm-level after accounting for the variables in the model. Thus, performing diagnostics on the model without the random effect was deemed to be acceptable. The final model included 11 covariate patterns. The Pearson χ^2 ^goodness-of-fit test was not significant (p = 0.12) indicating that we could not reject the null hypothesis that the model fit the data. There was one covariate pattern that had a standardized Pearson residual of 2.96 (deviance residual of 1.06). Ninety out of 212 flocks (42%) with this pattern were positive for *Campylobacter*, and the predicted probability of a positive flock was 39%. Flocks with this covariate pattern were raised in houses with vertical ventilation, the producer's boots were always cleaned and disinfected prior to entering the house, and geothermal water was used to clean the houses. Although this covariate pattern had the largest leverage (0.87) and delta-beta (58.1) values, it also had the highest number of observations (n = 212), thus, its moderate influence on the model was not of great concern.

Additionally, there was a covariate pattern with a standardized residual of -2.12 (deviance residual of -0.97). The observed percentage of positive flocks with this pattern was 38% (51 out of 133), and the predicted probability was 42%. Flocks with this covariate pattern were raised in houses with both vertical and horizontal vents, boots were always cleaned and disinfected prior to entering the house, and geothermal water was used to clean the houses. This covariate pattern had the second highest leverage value (0.79), delta-beta value (17.1), and number of observations (n = 133), thus, its influence on the model was of little concern.

## Discussion

Our study has identified four house-level risk factors associated with the colonization of broiler flocks with *Campylobacter *spp. in Iceland, out of a possible 30 explanatory variables. The higher ICC of a null model with a random effect at the farm level, compared to a null model with a random effect at the house level, suggests that there was more variation in flock *Campylobacter *status between farms than within farms. Thus, it was not unexpected that only a few house-level predictors remained statistically significant in the final model. In addition, our approach of screening variables within subsets enabled us to look at potentially complex relationships between predictors of a similar type, and identify those that were most strongly associated with flock status. For example, in some subsets (e.g. sanitation), including more than one predictor in the model led to unstable parameter estimates with high standard errors, therefore, only one predictor from the subset could be offered to the comprehensive model. Within other subsets, there was strong collinearity between variables, or between one variable and one category of another variable. For example, all six houses (contributing a total of 58 flocks) in which the roof and floor drains merged, also had roof drains that opened into a septic tank with overflow into an underground bed (Kendall's τ_b _= 1.0). In these subsets, only one of the two variables could be included in the analysis; the predictor resulting in the model with a smaller Akaike's Information Criteria (AIC) value was chosen. Additionally, there were several variables in which the responses were extremely one-sided; almost all producers cleaned the ventilation system after every flock and changed boots before entering the house, and all but one broiler house in Iceland have concrete floors (the floor of the upper level of one house included wood elements). These variables were eliminated from the analysis because it was unlikely that there would be enough power to detect such small differences.

Misclassification could have been a potential source of bias in our study. For some variables, it was necessary to combine categories if there were only a small number of observations in one or more categories. However, we expect this bias was small because we grouped only those categories that were biologically-related, and only when a Wald's test indicated that the categories were not statistically different from each other. In addition, eliminating predictors due to problems with collinearity or unstable parameter estimates might have affected our final results. However, we found that predictors that were strongly associated with flock status in the subset analyses, were also statistically significant in the final model. Thus, it is likely that our screening process was effective in identifying the most important of the house-level predictors.

For flocks with multiple catch lots, more pooled samples were obtained, and we considered whether this might have increased the likelihood that the flock would be positive for *Campylobacter*. In a previous analysis of flock-level factors [32], the number of catch lots was not a significant risk factor for the colonization of Icelandic broiler flocks with *Campylobacter*, after controlling for flock size. Most of the positive catch lots in our study were positive in all four pooled caecal samples, inferring high sensitivity of the methodology for the sample type. On a flock basis, 84% of the positive flocks yielded *Campylobacter *in all samples collected, and of the positive flocks with more than one catch lot, a high proportion were positive on the first catch lot. Several standard management practices in Iceland might have contributed to these findings. Beginning in 1999, before the start of the study period, the Icelandic poultry industry adopted a high standard of cleaning, chemical disinfection and drying of live haul crates and trucks, under the assumption that bringing crates covered with faecal material into broiler houses for partial depopulation was an important source of contamination to the remainder of the flock. Unlike other countries, Iceland does not have commercial catching crews that travel from farm to farm. The workers on each farm catch their own birds and follow their own biosecurity rules. In addition, the reason for collecting and slaughtering a flock in multiple catch lots differs in Iceland compared to other countries. In Iceland, the practice is based more on slaughter line speed capacity in the abattoirs; only the largest flocks (over 20,000 birds) are slaughtered in three or four catch lots, typically over three or four consecutive days. For flocks slaughtered in more than one catch lot, the average interval between catch lots is quite short. The practice of thinning (i.e. slaughtering half the flock as broilers and the second half as larger roasters) is rare in Iceland, and is typically done only during the Christmas season. During the winter, the prevalence of *Campylobacter *in broiler flocks is very low (see Methods). Therefore, because of the relatively short time interval between shipping catch lots, the low within-flock prevalence used in our sample size calculation to detect early stages of colonization, the high sensitivity of the isolation method, and the high proportion of catch lots that were positive in all samples, we expect that there was little variation with respect to the risk of false negative classification between flocks slaughtered in single and multiple catch lots.

Wild birds captured on broiler farm premises frequently carry *Campylobacter jejuni *[13,19,28,36-39]. *Campylobacter *prevalence has been shown to be higher on farms with frequent sightings of wild birds than on farms with few wild birds [12]. A previous study in the UK [16] has found that vertical ventilation systems are associated with positive flocks. In the present study, in an effort to determine if wild birds might be a source of *Campylobacter *to broiler flocks, we classified the ventilation system on each broiler house according to its type (vertical, horizontal, or both), and integrity (open or closed). An underlying assumption of our classification was that wild birds might be more likely to perch on vertical ventilation shafts, and, if those vents were open, the flock might be more likely to be exposed to the bacteria, either directly (e.g. wild birds entering the house or defecating into the vents), or indirectly (e.g. rain water washing faecal material in through the vents). From the subset analysis, we found that after controlling for ventilation type, the integrity of the ventilation system was not a significant risk factor for flock colonization (OR = 1.0, p = 0.975). However, the risk of colonization was predicted to increase when the flock was raised in a house with vertical, or vertical and horizontal ventilation shafts compared to horizontal vents. There are a few possible reasons for these findings. First, our classification of ventilation type might not have been refined enough (e.g. we did not differentiate between air inlets and outlets, and wild birds might preferentially perch on vertical outlets for warmth). Although researchers in Sweden [12] did not find an association between *Campylobacter *occurrence and air outlets (ceiling or wall) or inlets (passive ceiling, active ceiling, or active or passive wall vents), the authors stated that their conclusions might have been uncertain because confounding (univariable analysis only) and clustering were not accounted for in the analysis. Secondly, there might be some mechanism related to the type of ventilation system other than wild birds that contribute to flock colonization. Our finding of a protective effect of horizontal shafts might be related to the thoroughness of house sanitation, as it has been suggested that horizontal fans are more accessible for proper cleaning and disinfection [16]. A brief exploration of interactions between ventilation type and each sanitation variable revealed a significant positive interaction between vertical ventilation shafts and the use of bacterial disinfectant and/or fumigation, although, we did not include this interaction in the comprehensive model due to highly inflated and unreliable parameter estimates. Finally, the effect of ventilation type might be related to other potential sources of *Campylobacter *such as flies. Researchers in Denmark [40] have shown that flies captured within 50 m of a broiler house carried *Campylobacter*, and that per volume of ventilation air, 4.5 times the number of flies entered the house through a roof inlet than through wall inlets. Considering our findings, and those of Hald et al. (2004), further investigation in this area is warranted.

The risk of *Campylobacter *colonization was predicted to increase when the flock was raised in a house in which the producer's boots were cleaned and disinfected prior to entering the house. Using univariable analyses, several researchers [29-31,33] have not found an association between flock colonization and routines for cleaning and disinfecting footwear. Others [41] have noted an increasing percentage of *Campylobacter*-positive flocks with a decreasing hygiene score (including the disinfection of boots), although the hygiene score itself was not statistically significant. *Campylobacter jejuni *has been isolated from farmer's boots [13,19,22,39], and from a footbath containing tap water at the broiler house entrance [20]. Several studies in the UK [14,16,21] have shown that the effective use of boot dips prior to entering the houses either delayed or prevented colonization. Although our results were inconsistent with other studies, researchers in Sweden [12] noted that farmers were frequently careless about boot dipping (e.g. only dipping toes or heels, passing through the disinfectant very quickly, or dipping boots when clumps of mud were present). Thus, our results might reflect the improper use of boot dips by Icelandic producers, or, ineffective disinfectant solutions. An alternative explanation for the positive association might be related to the wording on our questionnaire. Producers were asked about cleaning and disinfection before entering the house (always, sometimes, or never). In retrospect, this question might not have been precise enough and we cannot be certain about what specific practices were represented or the consistency of recording. Nevertheless, the finding of an increased risk of colonization should encourage producers to assess the use and effectiveness of disinfectant boot dips, and their general cleaning and disinfection procedures prior to entering broiler houses.

Our study has shown that the risk of *Campylobacter *colonization was higher when the flock was raised in a house cleaned with geothermal (high temperature) water. The isolation of *Campylobacter *from surface water [20] and puddles [28] adjacent to broiler houses, points to possible environmental sources of *Campylobacter *to broiler flocks. Although not all geothermal wells were on-farm (i.e. some farms had geothermal water piped in), potentially, farm-based geothermal wells could have warm surface water pools, which might serve as *Campylobacter *reservoirs for birds, flies and other insects, and for the broiler flocks. However, several researchers [42-47] have found that isolation rates and/or survival of thermophilic *Campylobacter *spp. from various water sources were highest when the water temperature was between 2°C and 10°C, and lowest when the temperature exceeded 15°C. Thus, the reason for the positive association between *Campylobacter *status and the use of geothermal water for cleaning remains unclear. In Norway, the prevalence of *Campylobacter *in three surface water sources (lakes and rivers) was strongly associated with the number of indicator bacteria (from effluents, farming, and waterfowl) in the water [44]. Therefore, in our study, geothermal water might be a surrogate for farm location and associated environmental pressures for *Campylobacter *(e.g. proximity to *Campylobacter *reservoirs in cattle herds, broiler breeder and egg layer flocks, sheep, migratory waterfowl, or other environmental sources). To our knowledge, this is the first time this risk factor has been identified in the literature, and it will be of interest to ascertain if this predictor is recognized in future studies and to determine the mechanism of its effect.

## Conclusion

Our study has identified four risk factors related to broiler house attributes and house-management practices, for the occurrence of *Campylobacter *in broiler flocks in Iceland; vertical or vertical and horizontal ventilation shafts, cleaning and disinfecting boots prior to entering the broiler house, and the use of geothermal water for cleaning houses. We found that horizontal vents had a protective effect, therefore, extra care should be taken when cleaning vertical vents, and producers should ensure that the disinfectant used has an appropriate bactericidal effect for *Campylobacter*. Alternatively, the increased risk associated with vertical ventilation shafts might be related to the height of the vents and the potential for vectors such as flies to gain access to the house. This is an area that warrants further research, and further refinement in the classification of vertical and horizontal systems might be necessary for this purpose. For newly constructed houses, horizontal ventilation systems could be considered. Boot dipping procedures, including the frequency and thoroughness of dipping boots, the frequency of changing the dip, and the effectiveness of the dip against *Campylobacter *should be examined on farms experiencing a high prevalence of *Campylobacter*. Future studies are warranted to ascertain how geothermal water increases the risk of colonization, and to determine if this factor is a surrogate for micro-climatic conditions or agro-environmental pressures on the farm and surrounding region.

## Methods

### Target and study populations

The target population was commercial broiler chicken flocks raised in Iceland between May 2001 and September 2004. The study population included all broiler flocks produced by the three largest poultry companies in Iceland during the study period. In total, only 149 flocks (contributing less than 11% of the total broiler production in Iceland during the study period) from three farms in the north of Iceland and a coastal island were excluded, due to their remote location and associated difficulty in collecting data and samples.

### Data collection

Data on the characteristics of each farm and broiler house were gathered at the beginning of the study through a combination of phone interviews and site visits by the Veterinary Officer for Poultry Diseases of the Agricultural Agency of Iceland. Farms were also visited frequently during the study, and the recorded information was verified, including changes that occurred over time (e.g. major renovations, addition of new houses).

A questionnaire was used to collect house-level epidemiological data; this was administered by one of two field technicians of the Veterinary Officer for Poultry Diseases in a face-to-face interview with the producer after each flock was shipped for slaughter. Major subsets of variables included: ventilation; roof and floor drainage; building quality, materials and repair; house structure; pest proofing practices; biosecurity measures; sanitation practices; and house size. To ensure consistency in responses, data collected at the previous visit were reviewed with the producer, and any changes that occurred from one flock to the next were recorded.

Although the colonization of broiler flocks with *Campylobacter *is likely influenced by factors acting at more than one level of production (i.e. flock, house, farm, and possibly regional levels), and other factors potentially relevant to the epidemiology of *Campylobacter *were recorded, it was our intent in this study to restrict the analysis to factors acting specifically at the broiler-house level. Understanding the complex relationships between a large number of house-level management practices and broiler house characteristics (especially between factors of a similar type), and identifying the variable(s) from each major subset most strongly associated with flock *Campylobacter *status, was deemed necessary to direct interventions that might prevent *Campylobacter *from entering a broiler house (and thus prevent flock exposure). The group of factors chosen for this analysis were considered both sensible and comprehensive to satisfy the objectives of this study and were consistent with house-level factors reported in the literature.

### Bacteriological sampling and processing

Monthly reports summarizing records of flocks slaughtered each day were obtained from the processing plants. Flocks were collected and slaughtered in one to four catch lots depending on their size and on-farm management practices. At the processing plants, systematically selected caeca (including contents) were excised from 40 birds from each catch lot by the plant veterinarian and placed in sterile plastic bags to create four pooled samples containing ten caeca each. Samples were processed either the same day or after holding overnight at 4°C. The required sample size per flock was estimated to detect early stages of flock *Campylobacter *colonization or alleles with poor colonizing ability on the basis of a within-flock prevalence as low as 10%; four pooled samples would ensure 99% confidence of detecting at least one positive bird in a catch lot [48]. Serial dilutions of caecal contents were plated on Campy-Cefex agar [49] and incubated at 42°C under microaerobic conditions for 48 hours. In comparison to the NMKL method, which is the official method for *Campylobacter *isolation in Nordic countries, the Campy-Cefex direct plating method has a sensitivity and specificity of 97.8% and 97.6% (on a catch lot basis), and a sensitivity and specificity of 98.8% and 97.3% (on a pooled sample basis), respectively, for detecting *Campylobacter *spp. in broiler caecal samples at slaughter [50]. The Campy-Cefex method is used in the official Icelandic surveillance program because of its lower cost, shorter time for detection of *Campylobacter *spp., high sensitivity, and the ability to enumerate samples. Colonies were counted, and confirmed as *Campylobacter *spp. by microscopy and latex agglutination. A broiler flock was considered positive for *Campylobacter *if at least one of the pooled samples from any of the catch lots was positive on culture.

### Seasonal data

A summertime seasonal pattern of *Campylobacter *colonization of broiler flocks has been well-described in the literature. Over the full 3 1/2 year study period, 227 out of 1,425 flocks (15.9%) tested positive for *Campylobacter*. Almost all of the positive flocks (217 out of 227) were raised during the summer season (hatch dates between March 15 and September 15 of each year of the study). As a result of the strong seasonal variation in flock prevalence, it was of interest to focus our analysis on flocks raised during this high risk summer period. Our definition of summer corresponds to the periods of restrictions imposed by the Icelandic government on when manure is allowed to be spread on fields and pasture (March 15 to October 31).

### Definition of house-level variable

A house-level variable was considered to be a physical characteristic of the broiler house, or a management practice that was carried out at the house-level. We had initially assumed that these variables would be relatively consistent for all flocks raised in the same house during the study period. However, producers occasionally instituted changes, such that not all flocks raised in the same house were exposed to the same attribute or subjected to the same management practice. As a result, we were unable to collapse the data to the house level.

### Overview of statistical analysis

The data were analysed in two stages. Variables were initially screened (stage one) within logical subsets of risk factors (Tables 1 and 2). A multivariable analysis was used to screen the variables within each subset. Predictors with significant (p ≤ 0.15) conditional associations from each subset were identified and made available to a comprehensive model (stage two). Because the variables were screened within multivariable models, we chose a less liberal p-value than we would have if a univariable analysis had been utilized (e.g. p ≤ 0.25). We did not want to be too strict (e.g. p ≤ 0.05) during the screening stage, because: 1) most subsets contained only three or four variables; and 2) we were interested in offering a sufficient number of variables to the comprehensive model. In both stages, a backward elimination process was carried out and included the evaluation of correlations, confounding, and 2-way interactions between variables. For all models, we included a random effect at the farm-level to adjust for clustering using a latent variable method, based on the relatively high ICC of a null model with a farm random effect compared to a null model with a house random effect. The substantially higher variance of the farm random effect compared to the house random effect in a null model with random effects at both levels further confirmed that it was more important to control for clustering at the farm-level than at the house-level (see Results).

The process of variable selection was similar for all models and stages. For categorical predictors with more than two levels, a likelihood ratio test was used to assess the contribution of the variable to the model; for continuous and dichotomous predictors, a Wald's test was used. In addition, the AIC was used to compare non-nested models. As each variable was removed from a model, its effect on the coefficients of the other variables in the model was assessed. If the coefficient of another significant variable changed by more than 30%, the variable was deemed to be a confounder and was forced into the model. Once a main effects model was chosen, 2-way interaction terms were introduced one at a time and evaluated for statistical significance using either a Wald's test or likelihood ratio test. All statistical analyses were performed using Stata software version 8 (StataCorp, College Station, TX, USA).

### Variable screening using subset analyses (stage one)

Prior to modelling, frequency tables were used to scrutinize all categorical variables within each subset. Dichotomous predictors were excluded from the subset analysis if there were ≤ 10 observations in one category. For predictors with more than two levels, categories were combined if there were a small number of observations (≤ 15) in one or more categories, and/or to group biologically meaningful categories together.

In some cases, having two or more predictors in the same subset led to the occurrence of cells with very low frequencies of observations. For example, when a chemical cleaner was used to clean the house, but a bacterial disinfectant was not used, only three flocks were positive for *Campylobacter*. This led to very high standard errors of the parameter estimates. In these situations, the predictor with the strongest univariable association with *Campylobacter *status was made available to the comprehensive model.

Within each subset, correlations between predictors were evaluated (Kendall's τ_b _with adjustment for ties for categorical variables, and Pearson's correlation coefficient (r) for continuous variables). If one variable was collinear (τ or r ≥ 0.8) with another variable (or category of another variable) in the same subset, the predictor with the strongest univariable association was made available to the comprehensive model. If the models were not nested, the predictor resulting in the model with a smaller value of the AIC was made available.

Continuous predictors are summarized in Table 2. The assumption of a linear relationship between each continuous predictor and the outcome was evaluated using several methods. First, by dividing the predictor into equal categories, then plotting the log odds of the outcome against the category means of the predictor and visually assessing the linear relationship. Second, by generating a smoothed scatter plot of the probability of the outcome against the predictor and similarly evaluating the relationship. Third, by adding a quadratic term to the regression model and assessing its significance, with p ≤ 0.05 confirming a non-linear relationship.

### Comprehensive model (stage two)

Significant predictors from each subset (Tables 1 and 2) were made available to a comprehensive model. Main effects were chosen as described above, using a significance criterion of p ≤ 0.05 for inclusion in the model. Variables identified as confounders in the subset analyses were monitored for their effects on the coefficients of significant variables remaining in the comprehensive model. Due to the large number of predictors available for analysis, only those interactions that were both biologically sensible and did not substantially inflate the standard errors of the estimates (i.e. had low multicollinearity), were considered for inclusion in the final model.

Due to the limitations associated with assessing residuals and other diagnostics in a random effects model in Stata, diagnostics were performed on the model without the random effect. We used the Pearson χ^2 ^goodness-of-fit test to assess the overall fit of the model, with p ≤ 0.05 indicating a poor fit. Deviance and standardized Pearson residuals were calculated on the basis of one per covariate pattern; patterns with residuals less than or greater than 2.0 were examined. Potential influential observations were identified by examining large leverage and delta-beta [51] values.

## Authors' contributions

MTG performed the statistical analysis and drafted the manuscript. WM, OB and SAM critically evaluated the analysis and revised the manuscript for intellectual content. JR was involved in the conception, design and coordination of the study, data collection and data quality checks, and revision of the manuscript for intellectual content. JRB was involved in the conception and design of the study, the design of the epidemiological database structure, building the data query for the statistical analysis, and revision of the manuscript for intellectual content. RL was involved in the conception, design and coordination of the study, data management and final data quality control, and revision of the manuscript for intellectual content. All authors read and approved the final manuscript.
